# Temperature-Directed Reprogramming of Volatile and Semi-Volatile Metabolism in *Ginkgo biloba* Microclones Under Cold and Heat Stress

**DOI:** 10.3390/ijms27083393

**Published:** 2026-04-10

**Authors:** Nazym Korbozova, Lidiia Samarina, Elvira Shadenova, Dariga Dairbekova, Malika Yerbay, Nina Terletskaya

**Affiliations:** 1 Faculty of Biology and Biotechnology, Al-Farabi Kazakh National University, Al-Farabi 71/19, Almaty 050040, Kazakhstan; ddayrbekova@list.ru (D.D.); malika.isa99@mail.ru (M.Y.); 2 Institute of Genetics and Physiology, Al-Farabi Avenue 93, Almaty 050060, Kazakhstan; samarinalidia@gmail.com (L.S.); shadel08@mail.ru (E.S.)

**Keywords:** *Ginkgo biloba*, temperature stress, cold stress, heat stress, specialized metabolism, terpenoids, sesquiterpenes, oxy-sesquiterpenes, volatile metabolites, GC-MS, in vitro culture, metabolic reprogramming

## Abstract

Temperature is a major determinant of plant metabolic plasticity, yet its role in directing volatile and semi-volatile specialized metabolism in *Ginkgo biloba* remains poorly understood. In this study, we investigated how contrasting low- and high-temperature treatments reshape secondary metabolite contents in *G. biloba* microclones cultivated in vitro. Plants were exposed to cold (+3 °C) and heat (+30 °C) conditions, and their responses were analyzed using GC–MS profiling, anatomical measurements, chlorophyll fluorescence, and multivariate statistics. Cold treatment selectively increased the abundances of monoterpenes (13.22%) and sesquiterpenes (13.83%), with the strongest accumulation of caryophyllene, eucalyptol, and (1S)-camphor. In contrast, heat treatment reduced ester content to 3.73% and strongly enriched oxy-sesquiterpenes (46.50%) and lactone/ketone/spiroketone (29.54%) contents. The enhanced accumulation of isocalamendiol, isoshyobunone, cyclohexanone derivative, dehydroxy-isocalamendiol, and (+)-2-bornanone was observed under heat. According to the multivariate analysis, control plants were associated with traits reflecting optimal physiological performance, including greater parenchyma, phloem, and xylem thickness, larger vascular bundles, longer stomata, and higher NPQ, qN, Y(NPQ), and F_v_/F_m_. Cold-treated plants showed thicker epidermis and sclerenchyma, higher stomatal density and width, elevated Y(NO), and an enrichment of esters and terpenoids, whereas heat-treated plants were characterized by thicker adaxial and abaxial epidermis, increased mesophyll thickness, and higher levels of oxygenated metabolites. These findings expand current knowledge beyond terpene trilactones and flavonoids and identify *Ginkgo* microclones as a useful in vitro model for temperature-guided metabolic reprogramming and targeted metabolite enrichment.

## 1. Introduction

The temperature factor is among the most powerful environmental factors shaping plant metabolism. Both low and high temperatures perturb membrane properties, photosynthetic performance, redox homeostasis, carbon allocation, and the transcriptional regulation of stress-responsive biosynthetic pathways. As a consequence, temperature stress does not merely inhibit growth; it can reprogram specialized metabolism and alter both the abundance and composition of secondary metabolites, including volatile terpenoids and related oxygenated products [1,2]. In this context, temperature should be viewed not only as a damaging factor, but also as an important regulatory factor affecting metabolite accumulation. Recent metabolic modeling has demonstrated that the majority of secondary metabolite pathways are negatively coupled with plant growth objectives, with an approximately 3.9 × 10^−4^ decrease in biomass flux per unit increase in secondary metabolite production. The growth-differentiation balance hypothesis suggests that reducing resource availability from high to moderate levels decreases growth while potentially increasing secondary metabolism [3]. Among temperature-sensitive plant metabolites, terpenoids are especially relevant because their synthesis is directly linked to the plastidial methylerythritol phosphate (MEP) and cytosolic mevalonate (MVA) pathway, which is a crucial metabolic pathway in eukaryotes, archaea, and some bacteria that produces essential molecules, including cholesterol, isoprenoids, and dolichols. It begins with Acetyl-CoA and, via the rate-limiting enzyme HMG-CoA reductase, converts HMG-CoA into mevalonate, regulating cellular sterol levels. MVA pathways and are readily modulated by stress signaling, reactive oxygen species (ROS), calcium fluxes, and transcriptional regulators. Reviews focused on plant terpenes under environmental stress have shown that abiotic cues can strongly reshape both terpene biosynthesis and terpene emission, with stress-dependent shifts between constitutive blends and induced volatile profiles [4,5,6].

*G. biloba* is a valuable system in which to study such regulations. Besides its well-known flavonoids and terpene trilactones, *G. biloba* contains diverse volatile constituents, and published analyses of leaf volatiles indicate that sesquiterpenes can represent a substantial fraction of the volatile profile [7,8]. Physiological and transcriptomic analyses have revealed complex interactions between temperature stress and metabolic reprogramming in *G. biloba*, involving alterations in soluble sugars, proline, antioxidant enzymes, and hormone levels. Heat stress activates heat shock transcription factors (HSFs) and other regulatory networks, while cold and drought induce distinct transcriptional responses [9]. Studies under in vitro conditions have demonstrated increased production of secondary metabolites under various stress treatments, highlighting the potential for tissue culture systems to elucidate stress-induced metabolic shifts. In particular, cold was shown to increase total terpene trilactone content together with the expression of key biosynthetic genes, while environmental variation also reshapes flavonoid accumulation patterns in leaves [10,11]. Thus, the species is clearly metabolically plastic; however, the literature is still much less explicit about how contrasting low and high temperatures redirect the volatile/semi-volatile metabolite spectrum, especially the balance between sesquiterpene hydrocarbons and more oxygenated metabolite pools.

In other plant systems, chilling and heat have both been shown to alter volatile terpenoid biosynthesis, although the direction of change depends on species, tissue, and stress regime. Chilling can suppress or remodel fragrance-related volatile biosynthesis, whereas heat can induce large increases in terpenoid emissions or shift volatile output toward oxidized and stress-associated products [5,12,13,14,15,16]. Therefore, for metabolite classes such as sesquiterpenes, oxygenated terpenoids, ketones, and lactone-related compounds, temperature stress is best interpreted not as a single generic trigger of “defense”, but as a selective driver of biosynthetic redirection.

Plant tissue culture provides a powerful experimental platform for such analyses. In vitro systems allow precise control of temperature and other environmental variables, minimize confounding field effects, and enable reproducible assessment of stress responses at cellular and tissue levels [1,17,18].

From this perspective, *G. biloba* in vitro cultures offer a useful experimental platform for asking a more specific question than is usually posed in stress physiology: not simply whether temperature causes injury or activates protective responses, but whether contrasting thermal regimes can be used to steer the accumulation of chemically and potentially pharmacologically valuable metabolite groups. In the present study, special attention was therefore paid to temperature-dependent changes in terpenoid- and sesquiterpenoid-rich fractions, including oxygenated derivatives, as well as to the redistribution of metabolite classes toward ketone- and lactone-containing profiles under heat. We hypothesized that cold and heat would act as distinct temperature factors redirecting specialized metabolism in *G. biloba* microclones, generating different biosynthetic outputs rather than a single undifferentiated stress signature.

## 2. Results

### 2.1. Temperature Stress Reshaped the Secondary Metabolite Landscape of Ginkgo biloba In Vitro

**Figure 1:**
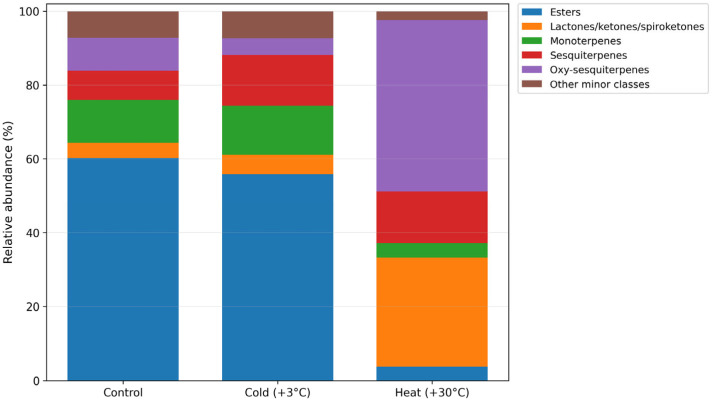
Temperature-driven redistribution of the major metabolite classes in *Ginkgo biloba* microclones cultured in vitro under control, cold (+3 °C), and heat (+30 °C) conditions. Stacked bars show the relative abundance (%) of the principal biochemical classes identified by GC–MS, including esters, lactones/ketones/spiroketones, monoterpenes, sesquiterpenes, and oxy-sesquiterpenes. The category “other minor classes” represents the remaining detected compounds not included in the five major groups. Cold treatment was associated with an increase in mono- and sesquiterpene fractions, whereas heat treatment caused a pronounced shift toward oxy-sesquiterpenes and lactone/ketone/spiroketone-rich metabolites, accompanied by a strong depletion of esters.

GC–MS profiling revealed pronounced temperature-dependent changes in the qualitative and semi-quantitative composition of secondary metabolites in *G. biloba* microclones cultivated in vitro. Across all treatments, compounds belonging to several major chemical classes were detected, including esters, monoterpenes, sesquiterpenes, oxy-sesquiterpenes, and lactones/ketones/spiroketones, together with other minor metabolite groups. However, the relative contribution of these classes differed substantially among control, cold-treated, and heat-treated plants, indicating a marked redirection of metabolic output under thermal stress (Figure 1).

Under control conditions, the metabolite profile was clearly dominated by esters, which accounted for 60.22% of the total detected metabolites. Monoterpenes, sesquiterpenes, and oxy-sesquiterpenes represented 11.66%, 7.93%, and 8.88%, respectively, whereas lactones/ketones/spiroketones contributed 4.11%. The remaining 7.20% corresponded to other minor metabolite classes. Thus, the control plants were characterized by an ester-rich metabolic state with a moderate contribution of terpenoid-related fractions.

Cold treatment altered this baseline profile, but the overall metabolic state remained ester-dominated. In cold-treated plants, esters still represent 55.82% of the total profile. At the same time, the contribution of monoterpenes increased to 13.22%, and sesquiterpenes increased to 13.83%, whereas oxy-sesquiterpenes decreased to 4.46%. Lactones/ketones/spiroketones remained low at 5.32%, and other minor classes accounted for 7.35%. These data indicate that low temperature did not cause a global metabolic redistribution but rather promoted a selective increase in mono- and sesquiterpene fractions within an otherwise ester-rich background.

In contrast, heat treatment induced the strongest reorganization of the metabolite profile. Under this condition, the ester fraction declined sharply to 3.73%, while oxy-sesquiterpenes became the dominant class, reaching 46.50% of the total detected metabolites. Lactones/ketones/spiroketones also increased markedly to 29.54%, whereas sesquiterpenes accounted for 13.97% and monoterpenes remained low at 3.90%. Other minor classes represented only 2.36% of the total profile. Thus, elevated temperature redirected metabolism toward a chemically distinct state dominated by oxygenated and structurally complex metabolites.

**Figure 2:**
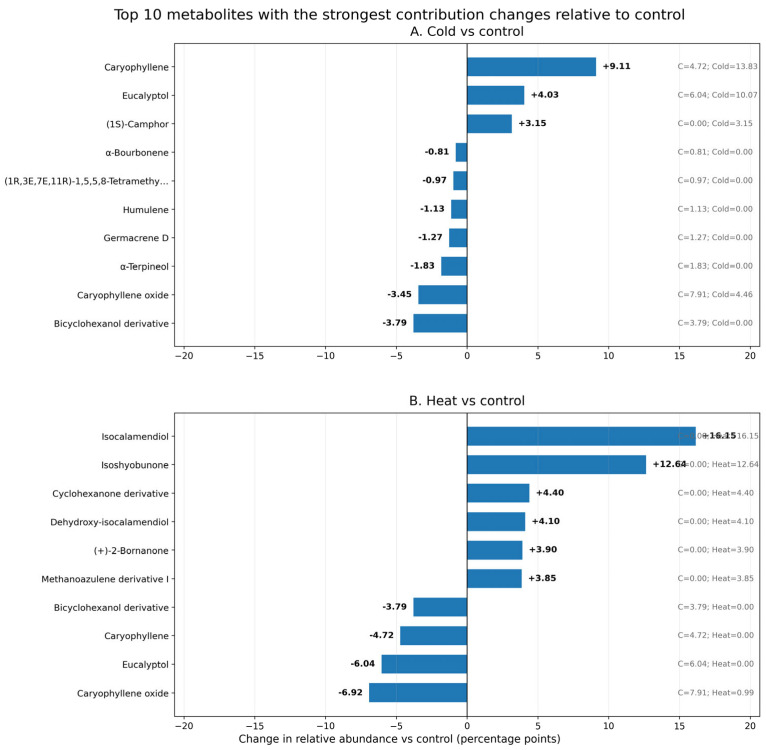
Top 10 individual metabolites showing the strongest changes in contribution to the overall metabolite profile of *Ginkgo biloba* microclones. (**A**)—cold treatment (+3 °C) relative to control. (**B**)—heat treatment (+30 °C). Bars represent the change in relative abundance compared with the control, expressed in percentage points (treatment−control). Positive values indicate an increased contribution of a metabolite to the total GC–MS profile under treatment, whereas negative values indicate a decreased contribution. The metabolites with the largest absolute changes under cold treatment were caryophyllene, eucalyptol, Bicyclo[3.1.0]hexan-2-ol, 2-methyl-5-(1-methylethyl)-, (1α,2β,5α)-, caryophyllene oxide, Bicyclo[2.2.1]heptan-2-one, 1,7,7-trimethyl-, (1S)-, α-Terpineol, germacrene D, humulene, (1R,3E,7E,11R)-1,5,5,8-Tetramethyl-12-oxabicyclo[9.1.0]dodeca-3,7-diene, and α-Bourbonene. The metabolites with the largest absolute changes under heat treatment were isocalamendiol, isoshyobunone, caryophyllene oxide, eucalyptol, caryophyllene, (2R,3R,6S)-6-Isopropyl-3-methyl-2-(prop-1-en-2-yl)-3-vinylcyclohexanone, dehydroxy-isocalamendiol, (+)-2-Bornanone, (3S,3aS,6R,8aS)-3,8,8-Trimethyl-7-methyleneoctahydro-1H-3a,6-methanoazulene, and Bicyclo[3.1.0]hexan-2-ol, 2-methyl-5-(1-methylethyl)-, (1α,2β,5α)-.

Cold treatment (+3 °C) did not produce a global shift toward a terpenoid-dominated metabolite profile but instead caused selective changes in the contribution of individual metabolites relative to the control (Figure 2A). The strongest positive contribution changes under low temperature were observed for caryophyllene (+9.11 percentage points), eucalyptol (+4.03 percentage points), and (1S)-camphor (+3.15 percentage points). In contrast, several metabolites decreased in relative contribution, including a bicyclohexanol derivative (−3.79 percentage points), caryophyllene oxide (−3.45 percentage points), α-terpineol (−1.83 percentage points), germacrene D (−1.27 percentage points), and humulene (−1.13 percentage points).

Exposure to elevated temperature (+30 °C) produced a metabolite profile clearly distinct from that of the control and cold-treated plants (Figure 2B). The strongest positive contribution changes under heat treatment were observed for isocalamendiol (+16.15 percentage points), isoshyobunone (+12.64 percentage points), cyclohexanone derivative (+4.40 percentage points), dehydroxy-isocalamendiol (+4.10 percentage points), (+)-2-bornanone (+3.90 percentage points), and methanoazulene derivative I (+3.85 percentage points). In contrast, several metabolites that contributed to the control profile were reduced under heat treatment, including caryophyllene oxide (−6.92 percentage points), eucalyptol (−6.04 percentage points), caryophyllene (−4.72 percentage points), and a bicyclohexanol derivative (−3.79 percentage points).

**Figure 3:**
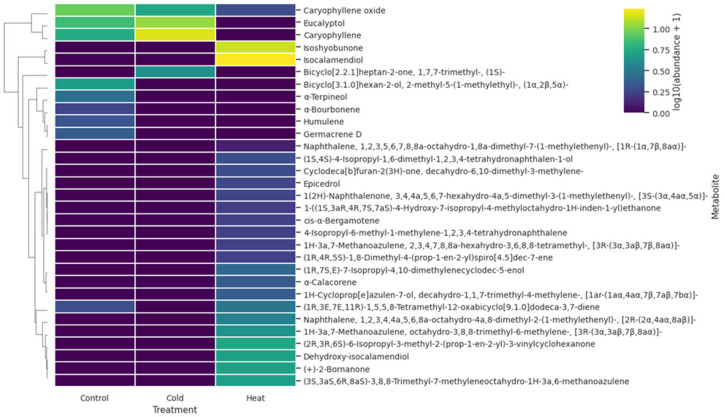
Heatmap showing treatment-level relative abundance values of volatile metabolites in *Ginkgo biloba* microclones under control, cold, and heat conditions. Because volatile compounds were quantified from pooled samples, the heatmap summarizes one chemical profile per treatment rather than replicate-level metabolite measurements. Values were log10(x + 1)-transformed for visualization. Rows are hierarchically clustered, whereas columns are kept in biological order.

Hierarchical clustering of treatment-level metabolite abundances further emphasized the distinct chemical outcomes induced by cold and heat stress (Figure 3). The heatmap resolved several metabolite modules with contrasting temperature associations. Cold-treated microclones were characterized by relatively higher abundances of caryophyllene, eucalyptol, and related terpenoid constituents, whereas heat-treated plants showed strong enrichment of oxygenated and structurally modified metabolites, including isocalamendiol, isoshyobunone, dehydroxy-isocalamendiol, (+)-2-bornanone, and several cyclohexanone- and azulene-related compounds. Control plants retained higher relative levels of some constitutive metabolites, including caryophyllene oxide, and showed an intermediate profile for several terpenoid compounds. Overall, the clustering pattern supports the conclusion that cold and heat did not induce a common metabolic response, but redirected secondary metabolism toward distinct treatment-specific chemical states.

### 2.2. Anatomical Remodeling and Chlorophyll Fluorescence Changes Defined the Physiological Context of Metabolic Reprogramming

**Figure 4:**
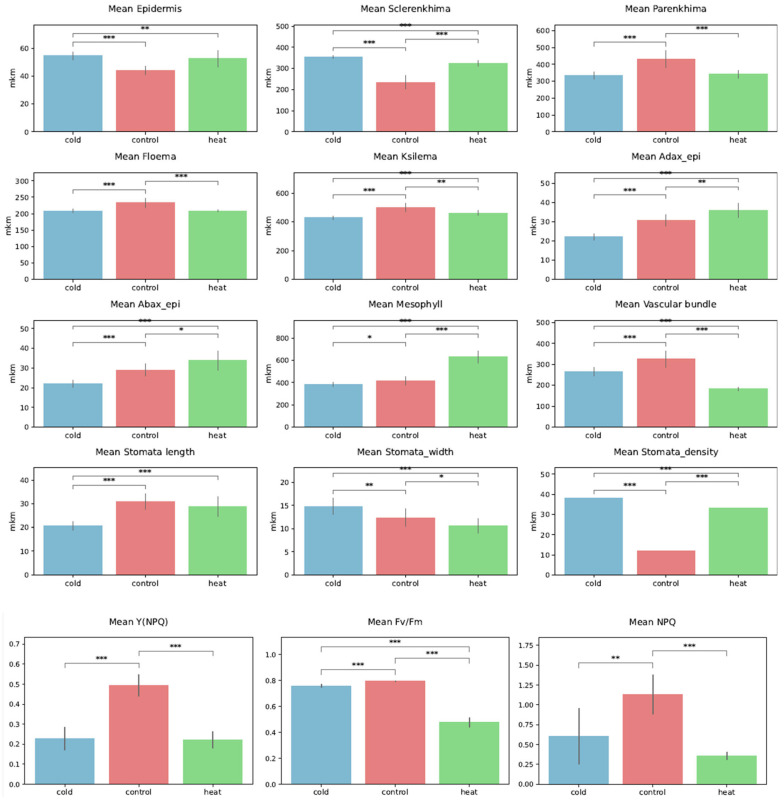
Effects of temperature treatments on anatomical traits and chlorophyll fluorescence parameters of *Ginkgo biloba* microclones cultivated in vitro. Bar plots show mean values ± SD for plants grown under cold, control, and heat conditions. Anatomical parameters include epidermis, sclerenchyma, parenchyma, phloem, xylem, mesophyll thickness, vascular bundle size, and stomatal traits (length, width, and density). Photosynthetic performance and photoprotective responses are represented by chlorophyll fluorescence parameters Y(NPQ), F_v_/F_m_, and NPQ. Statistically significant differences between treatments are indicated by asterisks (* *p* < 0.05, ** *p* < 0.01, *** *p* < 0.001).

Temperature stress induced pronounced and coordinated anatomical remodeling (Figure 4). Both cold and heat treatments resulted in a significant increase in epidermis thickness compared with the control. Its mean values were 54.49 μm (“cold”) and 52.46 μm (“heat”) as compared to 44.11 μm (“control”) (cold vs. control: *p* < 0.001 ***; heat vs. control: *p* < 0.01 **). Differences between “cold” and “heat” were not significant (*p* = 0.32).

A similar pattern was observed for sclerenchyma thickness, which increased markedly under both stress conditions, with the strongest response under cold treatment. The mean values were 354.06 μm (“cold”) and 324.61 μm (“heat”) as compared to 234.04 μm (“control”) (all pairwise comparisons highly significant, *p* < 0.001 ***). In contrast, “control” plants exhibited the largest parenchyma thickness (430.02 μm), significantly different from “cold” (333.74 μm) and “heat” (341.64 μm) (*p* < 0.001 ***).

Vascular tissues exhibited differentiated responses to temperature. Mesophyll thickness showed a strong increase under heat stress, suggesting structural acclimation of the photosynthetic tissue to elevated temperatures. Stomatal traits were strongly affected by temperature: stomatal density increased sharply under both cold (mean = 38.0) and heat (mean = 33.0) stress relative to control (mean = 12.0) (*p* < 0.001 ***). Conversely, stomatal length was significantly higher under control treatment and decreased toward cold and heat conditions.

Chlorophyll fluorescence parameters revealed substantial alterations in photosynthetic performance and photoprotective mechanisms. Control plants exhibited the highest values of NPQ and Y(NPQ), indicating more efficient regulated non-photochemical energy dissipation under optimal conditions. Particularly, NPQ (non-photochemical quenching) displayed a similar trend with the greatest value in “control” (1.130) as compared to “cold” (0.606) and “heat” (0.360) (control vs. cold: *p* < 0.01 **; control vs. heat: *p* < 0.001 ***). Additionally, Y(NPQ) (quantum yield of regulated energy dissipation) showed the greater value in “control” plants (0.494) as compared to “cold” (0.227) and “heat” (0.220) (*p* < 0.001 ***).

### 2.3. Multivariate Analysis Linked Temperature Treatments with Distinct Metabolic Signatures

**Figure 5:**
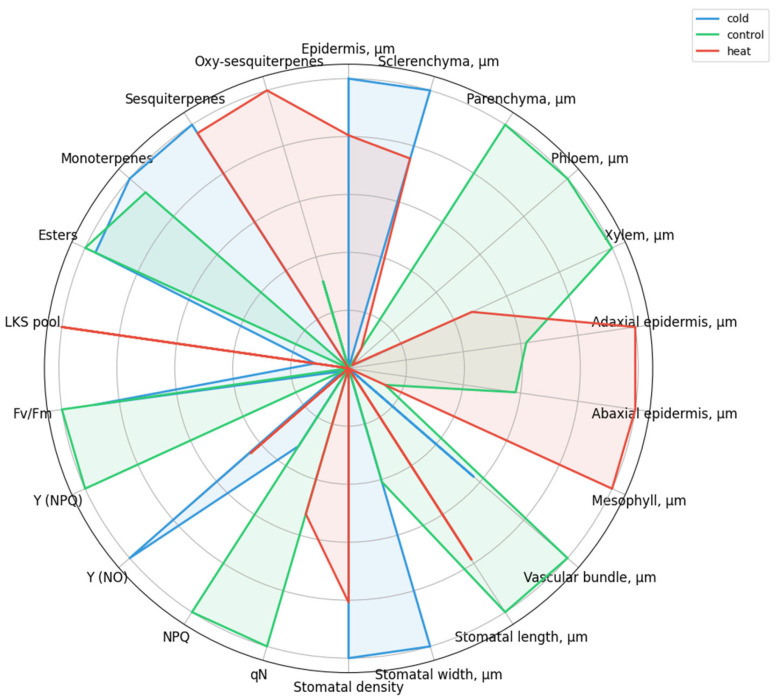
Spider plot showing scaled mean values of selected anatomical, chlorophyll fluorescence, and biochemical parameters of *Ginkgo biloba* microclones cultivated in vitro under cold, control, and heat treatments. Data are normalized using Min–Max scaling for visualization purposes; metabolic parameters are CLR-transformed. Each polygon represents the multivariate response profile of a treatment, illustrating relative shifts in structural traits, photosynthetic performance, and secondary metabolite composition under different temperature conditions.

The integration of anatomical, chlorophyll fluorescence, and biochemical traits further supported the existence of treatment-specific metabolic states. The spider plot highlights that control plants were characterized by relatively higher values of parameters associated with optimal physiological functioning, including parenchyma thickness, phloem and xylem development, vascular bundle thickness, stomatal length, NPQ, qN, Y(NPQ), and F_v_/F_m_ (Figure 5). Cold-treated plants exhibited a profile shift toward increased epidermis and sclerenchyma thickness, higher stomatal density and stomatal width, elevated Y(NO), and increased levels of esters, monoterpenes, and sesquiterpenes. At the same time, cold stress was associated with reduced NPQ, qN, and Y(NPQ), suggesting limitations in regulated photoprotective mechanisms. Heat-treated plants showed a contrasting response pattern, with pronounced increases in adaxial and abaxial epidermis thickness, mesophyll thickness, and elevated levels of the LKS pool, sesquiterpenes, and oxy-sesquiterpenes, indicating a shift toward a distinct structural and oxygenated metabolite-associated state.

**Figure 6:**
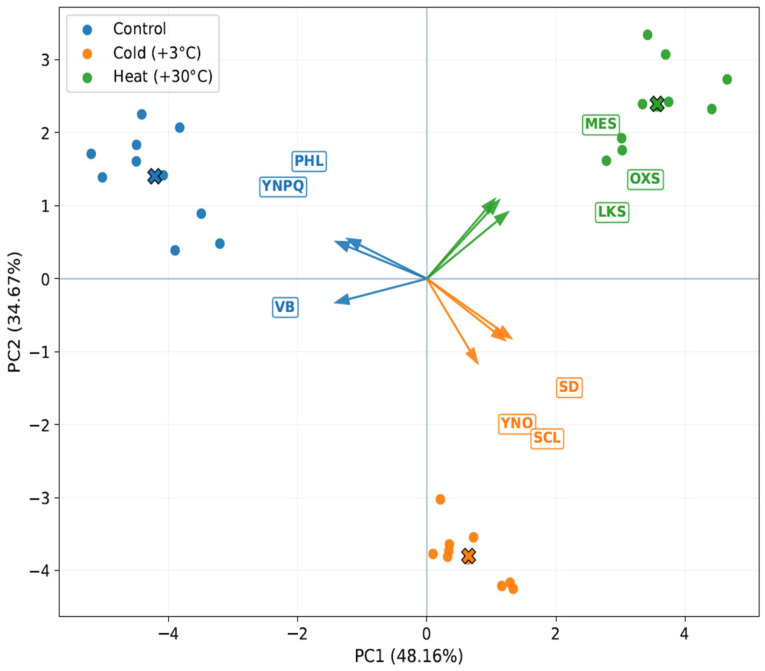
Principal component analysis (PCA) biplot showing the separation of control, cold-treated (+3 °C), and heat-treated (+30 °C) *Ginkgo biloba* microclones based on the integrated dataset including anatomical traits, chlorophyll fluorescence parameters, and major metabolite classes. Points represent individual samples, crosses mean centroids based on all data, whereas arrows indicate the selected variables whose loading vectors are most strongly aligned with the corresponding treatment clusters in the PC1–PC2 space. YNPQ = quantum yield of regulated non-photochemical energy dissipation in PSII, Y(NPQ); PHL = phloem thickness; VB = vascular bundle size; YNO = quantum yield of non-regulated energy dissipation in PSII, Y(NO); SD = stomatal density; SCL = sclerenchyma thickness; LKS = lactones/ketones/spiroketones; OXS = oxy-sesquiterpenes; MES = mesophyll thickness.

PCA results were consistent with those presented in the spider plot. The first two principal components explained a substantial proportion of the total variance, with PC1 accounting for 48.16% and PC2 for 34.67%, together capturing 82.83% of the total dataset variation (Figure 6). A clear separation of treatment groups was observed in the PC1–PC2 space. Control samples clustered on the negative side of PC1 and were associated mainly with Y(NPQ), phloem thickness, and vascular bundle size, indicating a stronger contribution of traits related to regulated photoprotective energy dissipation and vascular tissue development under non-stress conditions. In contrast, cold-treated samples were positioned on the positive side of PC1 and the negative side of PC2 and were most strongly associated with Y(NO), stomatal density, and sclerenchyma thickness. Heat-treated samples occupied the positive side of PC1 and the upper region of PC2 and were associated primarily with lactones/ketones/spiroketones, oxy-sesquiterpenes, and mesophyll thickness, reflecting a distinct response state characterized by both anatomical remodeling and marked shifts in metabolite composition.

Overall, the results demonstrate that temperature stress in *G. biloba* microclones cultured in vitro led to substantial redirection of secondary metabolism, but the direction of this shift depended strongly on the thermal regime. Under control conditions, the metabolome was dominated by esters. Cold stress induced the strongest increase in terpenoids and sesquiterpenoids, especially sesquiterpene hydrocarbons and their oxygenated derivatives. Heat stress, in contrast, promoted a different metabolic output characterized by the enrichment of oxygenated and structurally complex compounds, including ketones, lactones, and spiroketones. Anatomical remodeling altered chlorophyll fluorescence, and multivariate clustering analyses consistently showed that these metabolic outcomes formed part of broader treatment-specific response states.

## 3. Discussion

A key point that emerges from our integrated dataset is that the metabolic shift was embedded in anatomical and photosynthetic reorganization. Under cold treatment, epidermis and sclerenchyma thickened, stomatal density increased, stomatal length decreased, and Y(NO) increased while NPQ and Y(NPQ) declined. In physiological terms, this combination is compatible with a leaf state in which diffusional control is tightened, but regulated thermal dissipation is weaker and a larger fraction of excitation energy is lost through non-regulated pathways. Because stomatal density and stomatal size together determine the potential upper limit of stomatal conductance, whereas mesophyll structural traits constrain internal CO_2_ diffusion and carbon gain, such a structural phenotype would be expected to alter both carbon availability and the redox context in which specialized metabolism operates [5,19]. Our cold-treated plants did not show a generalized rise in all terpenoid-derived metabolites; instead, they accumulated a narrower set of monoterpene and sesquiterpene hydrocarbons and relatively fewer oxygenated products. This pattern is compatible with a shift toward less extensively oxygenated volatile terpenes under cold treatment; however, any physiological interpretation of this shift is tentative now and based on an analogy from the literature [5,10,20,21].

Because the present study did not quantify ROS accumulation, lipid peroxidation, membrane stability, antioxidant enzyme activities, or signaling outputs, the detected metabolites we interpreted here primarily serve as correlates of the observed anatomical and photophysiological states, not as demonstrated causal agents.

Importantly, stability at the level of overall composition may mask active internal turnover, where carotenoid-based terpenoids undergo rapid cycling within NPQ processes without changing the total pool size detectable by endpoint metabolomics, underscoring why aligning such chemical measurements with chlorophyll fluorescence data is essential for functional interpretation.

Low and high temperature did not reinforce a single generic stress-associated metabolic program in *G. biloba* microclones; instead, they were associated with two chemically distinct output states. Cold treatment retained an ester-dominated metabolic background but selectively increased the contribution of several terpenoid constituents, most notably caryophyllene, eucalyptol, and (1S)-camphor, whereas heat treatment caused a much stronger redistribution of the metabolite profile toward oxy-sesquiterpenes and the lactone/ketone/spiroketone pool. This distinction is consistent with the broader literature showing that temperature stress can reprogram plant metabolism through coordinated effects on membrane properties, redox balance, carbon allocation, and downstream modification of specialized metabolites, rather than acting as a simple on/off inducer of “stress compounds” [2,4,22]. On that basis, a tentative literature-based interpretation of the cold-enriched compounds can be proposed. Caryophyllene, eucalyptol, and (1S)-camphor are relatively mobile lipophilic terpenoids, and their enrichment under low temperature may be consistent with a comparatively less oxidized volatile profile rather than with deep oxidative remodeling. However, in the absence of direct measurements of membrane integrity, oxidative damage, or signaling activity, we interpret these compounds conservatively as markers associated with the cold-treated state rather than as proven mediators of membrane protection or stress signaling [4,5,20,21].

The heat response revealed a different structure–metabolite relationship. Heat increased mesophyll thickness together with epidermal thickening and high stomatal density, but photosynthetic photoprotection remained depressed relative to the control, as indicated by the low NPQ and Y(NPQ) values. At the same time, the metabolome shifted decisively toward oxy-sesquiterpenes and the lactone/ketone/spiroketone pool. Such coupling is biologically meaningful at the level of correlation. Mesophyll architecture is a major determinant of mesophyll conductance and chloroplast exposure to intercellular air space, and therefore of the balance between CO_2_ assimilation, photorespiratory pressure, and excess reducing power [20,23]. By contrast with the cold effect, the metabolites that predominated under heat, including isocalamendiol, isoshyobunone, dehydroxy-isocalamendiol, (+)-2-bornanone, and cyclohexanone-related compounds, may tentatively indicate stronger oxidative tailoring of terpene scaffolds under heat-associated redox imbalance. Although the specific physiological role of each individual compound in Ginkgo cannot be assigned directly from the present data, their collective enrichment is consistent with a more oxygenated metabolite profile associated with the heat-treated state [5,8,20,21].

When this balance is perturbed by heat, the biosynthetic system may be driven not only toward terpene scaffold formation, but also toward oxidative tailoring reactions. In the framework of current terpenoid network models, this would correspond to a shift from primary branch flux into hydrocarbon products toward downstream oxygenation and structural rearrangement of sesquiterpene backbones [21]. The accumulation of isocalamendiol, isoshyobunone, dehydroxy-isocalamendiol, and cyclohexanone-type compounds in our heat-treated cultures can therefore be interpreted as a candidate metabolic signature of a heat-associated oxidative branch state, rather than as direct evidence of function. In parallel, the sharp depletion of esters indicates that heat was accompanied by a rebalancing of the relative metabolite profile away from the constitutive background pattern and toward a more oxygenated chemical composition. Such behavior agrees with general models of abiotic-stress photosynthesis in which reduced photochemical efficiency, altered energy dissipation, and ROS-related signaling are associated with secondary metabolic remodeling [20].

The anatomical data further refine this interpretation. Increased sclerenchyma under cold suggests reinforcement of mechanically and hydraulically conservative tissue states, whereas increased mesophyll under heat points to remodeling the photosynthetically active compartment itself. Importantly, stomatal densification in both stress treatments should not be interpreted automatically as improved carbon gain. Higher stomatal density combined with shorter stomata can increase theoretical diffusional capacity, but actual photosynthetic benefits depend on stomatal aperture dynamics, mesophyll conductance, and biochemical demand for CO_2_ [19,23]. In our experiment, the simultaneous decline in NPQ/Y(NPQ) and the rise in Y(NO) under stress indicate that the anatomical shifts did not preserve the control-like photophysiological state. Instead, they likely changed the balance between carbon assimilation, leaf cooling, and oxidative load, thereby helping determine which metabolite classes accumulated. This interpretation is important because it places the observed GC-MS shifts into a whole-leaf framework rather than treating them as isolated biochemical outputs.

This integrated view is also supported by recent work within *Ginkgo* itself. In a 2024 study of trumpet-shaped versus normal leaves, Li et al. combined tissue structure, photosynthetic traits, metabolomics, and transcriptomics and showed that altered epidermal asymmetry, stomatal distribution, photosynthetic performance, and secondary metabolism are tightly linked in *G. biloba* [24]. Although that study addressed developmental leaf-form variation rather than temperature stress, it is highly relevant here because it demonstrates within the same species that anatomical patterning, photosynthetic behavior, and metabolite composition are coordinated rather than independent layers of the phenotype. Likewise, Chang et al. showed that heat stress in *Ginkgo* triggers coupled physiological and metabolic responses, including ROS-related changes and metabolite remodeling [8]. Our results extend those observations from broad stress physiology to the volatile/semi-volatile metabolome and indicate that the anatomical–photophysiological context helps explain why cold and heat arrive at different metabolite endpoints.

From a biosynthetic standpoint, the most plausible mechanistic interpretation is that temperature altered both precursor allocation and the depth of downstream oxidation. The MEP and MVA pathways draw on photosynthesis-derived carbon skeletons, ATP, and reductant, and their outputs are coordinated through multilayer regulation and compartmental crosstalk [21]. Consequently, changes in photochemical energy use and CO_2_ diffusion are expected to propagate into terpenoid metabolism. Under cold, the phenotype observed here is compatible with selective enrichment of mono- and sesquiterpene hydrocarbons and only limited oxidative conversion; under heat, the data support stronger routing into oxygenated sesquiterpenes and ketone/lactone-rich products. We therefore infer that the decisive control point is unlikely to be total terpene pathway activation alone. Rather, it is the coupling of anatomical acclimation and photosynthetic state with branch-specific terpene tailoring reactions that defines the final metabolite spectrum. This conclusion also explains why the cold and heat profiles were not mirror images of one another, but qualitatively different biochemical states.

The practical implication is that anatomical and chlorophyll fluorescence traits can serve not only as descriptors of stress injury, but also as predictors of metabolite output during temperature elicitation. A cold-induced phenotype characterized by thicker protective tissues, denser but shorter stomata, reduced regulated quenching, and elevated Y(NO) was associated here with caryophyllene-, eucalyptol-, and camphor-enriched chemistry. A heat-induced phenotype characterized by mesophyll remodeling and depressed photoprotective dissipation was associated with oxygenated sesquiterpene- and ketone/lactone-rich chemistry. Thus, in vitro temperature treatments appear to steer Ginkgo metabolism through an integrated structure–photosynthesis–metabolome axis. Future work should test this model directly by combining time-resolved gas exchange, mesophyll conductance estimates, ROS profiling, and targeted quantification of key terpene intermediates and oxidized end products [8,20,21,24].

## 4. Materials and Methods

### 4.1. Plant Material and In Vitro Culture Conditions

The study was performed using microclonal plants of *Ginkgo biloba* L. maintained under aseptic in vitro conditions. Microclonal plants of *G. biloba* were established from donor trees growing in the Botanical Garden of Almaty, Kazakhstan. The trees were introduced from China in 1983 and have since been maintained as part of the living woody plant collection of the garden, which serves as a scientific base for plant introduction and acclimatization studies in the foothill zone of the Trans-Ili Alatau. Explants were surface-disinfected by sequential immersion in 70% ethanol for 30 s followed by sodium hypochlorite solution, 1–2% active chlorine, for 10–15 min, and then rinsed three times with sterile distilled water. Only viable, contamination-free explants were transferred to culture medium.

Explants were cultivated on Murashige and Skoog (MS) basal medium containing standard macro- and micronutrients [25]. The medium was supplemented with auxins included indole-3-acetic acid (IAA) 0.5 mg/L, IBA 0.1 mg/L and 2,4-dichlorophenoxyacetic acid (2,4-D) 1.0 mg/L, while cytokinins included 6-benzylaminopurine (BAP) 0.5mg/L and kinetin 1.0 mg/L, solidified with agar, 7 g L^−1^ and adjusted to pH 5.7 ± 0.1 before autoclaving. Cultures were maintained in a growth chamber under a 16 h light/8 h dark photoperiod at a photon flux density of 40–50 µmol m^−2^ s^−1^ provided by cool-white fluorescent lamps and at 25 ± 1 °C [26,27]. Microclones of the same developmental stage and similar size were selected for the experiment to minimize ontogenetic variation. The use of MS medium and aseptic in vitro culture conditions follows established plant tissue culture practice and is consistent with previous reports on *G. biloba* micropropagation.

### 4.2. Experimental Design and Temperature Stress Treatments

After 45 days of cultivation under standard conditions, morphologically uniform microclones were randomly assigned to three experimental groups: control, cold stress, and heat stress. A control group was grown under 25 ± 1 °C at day and 20 ± 1 °C at night. For cold treatment, culture vessels were transferred to a climate-controlled chamber set at +3 ± 1 °C, whereas heat treatment was imposed at +30 ± 1 °C. All groups were maintained under the same illumination intensity at 160–200 mmol m^−2^ s^−1^, with a 16 h photoperiod, in order to exclude confounding effects of light conditions. The duration of the temperature treatment was 72 h, selected to induce measurable anatomical, physiological, and biochemical responses without causing irreversible tissue necrosis.

**Figure 7:**
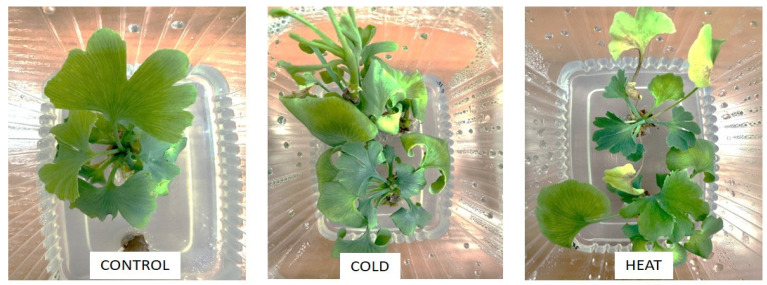
Experimental microclones of *G. biloba* at control, cold-treated (+3 °C), and heat-treated (+30 °C) conditions.

At the end of the exposure period, whole microclones were sampled for anatomical, chlorophyll fluorescence, and biochemical analyses. Each treatment included three independent biological replicates, with one culture vessel regarded as one biological replicate; each vessel contained 3–5 microclones (Figure 7). For destructive analyses, 3 plants per replicate were pooled.

### 4.3. Anatomical and Microstructural Analysis

Leaf and stem fragments collected from the middle part of the microclones were fixed in 70% ethanol and preservative fluid was a Strasburger–Fleming’s mixture: 96% etha-nol:glycerol:water in ratio of 1:1:1 [28]. The material was infused for a 24 h. Anatomical specimens were prepared with a microtome MZP-01 (“Technom”, Ekaterinburg, Russia) with a freezing unit OL-ZSO 30 (“Inmedprom”, Yaroslavl, Russia). The thickness of anatomical sections varied between 10 and 15 microns. The sections were placed on a glass slide in a drop of pure glycerin and covered with a cover slip to obtain temporary preparation. Micrographs of anatomic sections were made on a microscope with Micro Opix MX 700 (T) (West Medica, Brown Boveri-Straße 6, B17-1 2351 Wiener Neudorf, Austria), CAM V1200C HD-camera (West Medica, Brown Boveri-Straße 6, B17-1 2351 Wiener Neudorf, Austria). All anatomical data were obtained in 3 replicates (3 plants in each) and 5–7 fields of view per section were analyzed with a 40× objective and averaged before statis-tical analysis. Sample preparation and measurement procedures followed standard plant microtechnique and microscopy practice [29].

### 4.4. Chlorophyll a Fluorescence Measurements

Photosynthetic performance was assessed by chlorophyll fluorescence measurement on intact leaves of in vitro-grown microclones using a pulse-amplitude-modulated fluorometer JuniorPAM. Prior to measurement, samples were dark-adapted for 30 min to allow complete oxidation of PSII electron acceptors. Minimum fluorescence (F_0_) was determined under weak modulated measuring light, and maximum fluorescence (F_m_) was induced by a saturating pulse. The maximum quantum efficiency of PSII was calculated as F_v_/F_m_ = (F_m_ − F_0_)/F_m_. Under actinic illumination, the following parameters were determined: non-photochemical quenching (NPQ), coefficient of non-photochemical quenching (qN), effective quantum yield of regulated non-photochemical energy dissipation Y(NPQ), and quantum yield of non-regulated energy dissipation Y(NO) [30,31].

### 4.5. Biochemical Analysis of Secondary Metabolites

Gas chromatography with mass spectrometric detection (Agilent 6890 N/5973 N, Santa Clara, CA, USA) was used for determination of organic compounds. For this experiment, plant tissue samples were fixed in 96% ethanol at a ratio of 100 g of tissue: 500 mL ethanol. The extraction was carried out in an orbital shaker in two stages (72 h each) with the same solvent until a clear colorless solvent was obtained. Sample volume was 1.0 µL, sample injection temperature was 260 °C, without flow division. Each sample was injected into the GC-MS system one time (three technical repetitions in total). Separation was carried out using a chromatographic capillary column DB-35 MS with a length of 30 m, an inner diameter of 0.25 mm, and a film thickness of 0.25 µm at a constant carrier gas (helium) velocity of 1 mL/min. The chromatographic temperature was programmed from 40 (exposure 0 min) to 150 °C with a heating rate of 10 °C/min (exposure 0 min) and up to 300 °C with a heating rate of 5 °C/min (exposure 10 min). Detection was carried out in the SCAN *m*/*z* 34–850 mode. Agilent MSD ChemStation software (version 1701EA) (Santa Clara, CA, USA) was used to control the gas chromatography system and to register and process the obtained results and data. For data processing, the average values of the obtained data were taken. Data processing included determination of retention times and peak areas, as well as processing. Relative abundance was estimated by peak-area normalization and expressed as the percentage of the total ion current/total ion chromatogram area. Because these values represent compositional data, they were used as relative rather than absolute concentrations [32,33,34]. Spectral information was obtained using a mass spectrometric detector. The Wiley 7th edition and NIST’02 libraries were used to decode the obtained mass spectra (the total number of spectra in the libraries is more than 550 thousand).

### 4.6. Statistical and Multivariate Data Analysis

All statistical analyses were performed in Python, version version 3.14.4. For anatomical and chlorophyll fluorescence variables, data distribution was assessed using the Shapiro–Wilk test and homogeneity of variances was evaluated prior to between-treatment comparisons. For variables satisfying parametric assumptions, differences among control, cold, and heat treatments were tested by one-way analysis of variance (ANOVA) followed by Tukey’s HSD post hoc test. When homogeneity of variances was not met, Welch’s ANOVA followed by Games–Howell multiple comparisons was applied. Results are presented as mean ± standard deviation (SD).

For multivariate analyses, an integrated data matrix was assembled from anatomical traits, chlorophyll fluorescence parameters, and major metabolite-class variables. One culture vessel was treated as one biological replicate; for destructive biochemical analyses, pooled plants from the same replicate yielded one replicate-level metabolite profile. Because metabolite-class data were compositional, these variables were CLR-transformed before multivariate integration.

For the spider plot, treatment mean values were calculated for each selected variable. Anatomical and chlorophyll fluorescence parameters were normalized by Min–Max scaling for visualization, and the transformed metabolite variables were plotted on the same comparative scale. The spider plot was used as a descriptive visualization of relative multivariate response profiles and was not used for inferential testing.

For principal component analysis (PCA), the replicate-level integrated data matrix was autoscaled by mean-centering and division by the standard deviation of each variable. PCA was then performed on the standardized dataset, and the first two principal components were visualized as a biplot. Sample scores were used to display treatment separation, while variable loadings were used to indicate the traits most strongly associated with each treatment group.

## 5. Conclusions

Cold and heat stress were associated with distinct metabolite profiles in *G. biloba* microclones rather than a common response. Cold treatment maintained an ester-dominated profile but selectively increased caryophyllene, eucalyptol, and (1S)-camphor, whereas heat was associated with redistribution toward oxy-sesquiterpenes and lactones/ketones/spiroketones, with isocalamendiol and isoshyobunone showing the largest positive shifts. Multivariate analysis further showed that these biochemical patterns coincided with distinct anatomical and photophysiological states. Although the present data support correlation with physiological states rather than direct causation, it can be argued that the temperature is an important factor associated with volatile and semi-volatile metabolic reprogramming in *G. biloba* under in vitro conditions.

## Data Availability

The original contributions presented in this study are included in the article. Further inquiries can be directed to the corresponding authors.
